# 9-Ethyl-3,6-diformyl-9*H*-carbazole

**DOI:** 10.1107/S1600536808017923

**Published:** 2008-06-19

**Authors:** Jun Jie Wang, Xian Zhang, Bao Qin Zhang, Gang Wang, Xiao Qiang Yu

**Affiliations:** a State Key Laboratory of Crystalline Materials, Institute of Crystalline Materials, Shandong University, Jinan 250100, People’s Republic of China; b School of Materials Science and Engineering, Shandong Institute of Light Industry, Jinan 250353, People’s Republic of China; c School of Chemistry and Chemical Engineering, Shandong University, Jinan 250100, People’s Republic of China

## Abstract

The structure of the title compound, C_16_H_13_NO_2_, was determined as a part of a project on the synthesis of new compounds which can make two-photon absorptions. In the crystal structure, both aldehyde groups are located within the carbazole plane. One of these groups is disordered and was refined using a split model with site-occupation factors for each position of 0.5.

## Related literature

For the synthesis of 9-ethyicarbazole, see: Li *et al.* (2001 ▶).
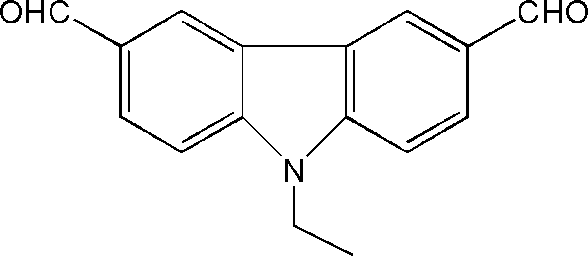


## Experimental

### 

#### Crystal data

C_16_H_13_NO_2_
*M* *_r_* = 251.27
Monoclinic, 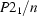

*a* = 13.5475 (3) Å
*b* = 6.69540 (10) Å
*c* = 14.1840 (2) Å
β = 100.5100 (10)°
*V* = 1264.99 (4) Å^3^
*Z* = 4
Mo *K*α radiation
μ = 0.09 mm^−1^
*T* = 293 (2) K
0.46 × 0.32 × 0.28 mm

#### Data collection

Bruker APEX2 CCD area-detector diffractometer
Absorption correction: multi-scan (*APEX2*; Bruker, 2005 ▶) *T*_min_ = 0.962, *T*_max_ = 0.978
7890 measured reflections
2810 independent reflections
2123 reflections with *I* > 2σ(*I*)
*R*_int_ = 0.016

#### Refinement

*R*[*F* ^2^ > 2σ(*F* ^2^)] = 0.038
*wR*(*F* ^2^) = 0.112
*S* = 1.05
2810 reflections
183 parameters
H-atom parameters constrained
Δρ_max_ = 0.17 e Å^−3^
Δρ_min_ = −0.16 e Å^−3^

### 

Data collection: *APEX2* (Bruker, 2005 ▶); cell refinement: *APEX2*; data reduction: *APEX2*; program(s) used to solve structure: *SIR97* (Altomare *et al*., 1999 ▶); program(s) used to refine structure: *SHELXL97* (Sheldrick, 2008 ▶); molecular graphics: *SHELXTL* (Sheldrick, 2008 ▶); software used to prepare material for publication: *WinGX* (Farrugia, 1999 ▶).

## Supplementary Material

Crystal structure: contains datablocks I, global. DOI: 10.1107/S1600536808017923/nc2104sup1.cif

Structure factors: contains datablocks I. DOI: 10.1107/S1600536808017923/nc2104Isup2.hkl

Additional supplementary materials:  crystallographic information; 3D view; checkCIF report

## Figures and Tables

**Fig. 1.**
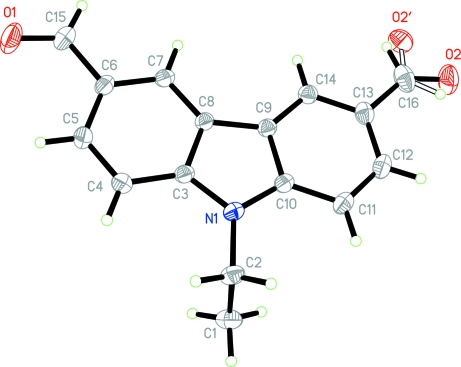
: The molecular structure of title compound with labelling and 50% probability displacement ellipsoids.

## supplementary crystallographic information

### Experimental

9-Ethylcarbazole was synthesized according to the literature (Li *et al. *, 2001). Anhydrous DMF (22 mL, 0.3 mol) was added dropwisely to POCl_3_ (28 ml, 0.3 mol) under stirring in an ice bath.  After 30 minute a white precipitate is obtained and a solution of 9-ethylcarbazole (3.155 g, 16 mmol) in DMF (20 mL) were added. The reaction mixture was slowly heated to 373 K and stirred at this temperature for 30 h and then cooled to room temperature. The brown viscous oily production was poured into the ice-water and shaken; the pH value of the solution was adjusted to 8 by dropping aqueous sodium hydroxide and sodium bicarbonate. It was stirred for another 2 h at pH=8 at room temperature. The aqueous layer was extracted with dichloromethane (3 × 100 ml) and the combined organic layers were washed three times with 100 mL of water and dried over anhydrous magnesium sulfate. Afterwards the solvent was evaporated under reduced pressure. The residue was dissolved in a minimal amount of dichloromethane and then purified by silica-gel column chromatography using dichloromethane as eluent. The product was recrystallized from dichloromethane to give high quality dark yellow crystals used for X-ray structure analysis.

### Refinement

All H atoms were placed in geometrically calculated positions and refined using a riding model with C—H = 0.97 Å (for CH_2_ groups) and 0.96 Å (for CH_3_ groups). Their isotropic displacement parameters were set to 1.2 times (1.5 times for CH_3_ groups) of the equivalent displacement parameter of their parent atoms. The O2 oxygen atom is disordered over two positions and was refined using a split model with half occupancy for each site.

### Crystal data

**Table:**

C_16_H_13_NO_2_	*F*_000_ = 528
*M**_r_* = 251.27	*D*_x_ = 1.319 Mg m^−^^3^
Monoclinic, *P*2(1)/*n*	Mo *K*α radiation λ = 0.71073 Å
*a* = 13.5475 (3) Å	Cell parameters from 3121 reflections
*b* = 6.69540 (10) Å	θ = 2.9–27.0º
*c* = 14.1840 (2) Å	µ = 0.09 mm^−^^1^
β = 100.5100 (10)º	*T* = 293 (2) K
*V* = 1264.99 (4) Å^3^	Block, colourless
*Z* = 4	0.46 × 0.32 × 0.28 mm

### Data collection

**Table:**

Bruker APEX2 CCD area-detector diffractometer	2810 independent reflections
Radiation source: fine-focus sealed tube	2123 reflections with *I* > 2σ(*I*)
Monochromator: graphite	*R*_int_ = 0.016
*T* = 293(2) K	θ_max_ = 27.5º
φ and ω scans	θ_min_ = 1.9º
Absorption correction: multi-scan(APEX2; Bruker, 2005)	*h* = −15→17
*T*_min_ = 0.962, *T*_max_ = 0.978	*k* = −8→8
7890 measured reflections	*l* = −18→18

### Refinement

**Table:**

Refinement on *F*^2^	Hydrogen site location: inferred from neighbouring sites
Least-squares matrix: full	H-atom parameters constrained
*R*[*F*^2^ > 2σ(*F*^2^)] = 0.038	*w* = 1/[σ^2^(*F*_o_^2^) + (0.0515*P*)^2^ + 0.1926*P*] where *P* = (*F*_o_^2^ + 2*F*_c_^2^)/3
*wR*(*F*^2^) = 0.112	(Δ/σ)_max_ = 0.001
*S* = 1.05	Δρ_max_ = 0.17 e Å^−^^3^
2810 reflections	Δρ_min_ = −0.16 e Å^−^^3^
183 parameters	Extinction correction: SHELXL97 (Sheldrick, 1997), Fc^*^=kFc[1+0.001xFc^2^λ^3^/sin(2θ)]^-1/4^
Primary atom site location: structure-invariant direct methods	Extinction coefficient: 0.020 (3)
Secondary atom site location: difference Fourier map	

### Special details

**Table:**

Geometry. All e.s.d.'s (except the e.s.d. in the dihedral angle between two l.s. planes) are estimated using the full covariance matrix. The cell e.s.d.'s are taken into account individually in the estimation of e.s.d.'s in distances, angles and torsion angles; correlations between e.s.d.'s in cell parameters are only used when they are defined by crystal symmetry. An approximate (isotropic) treatment of cell e.s.d.'s is used for estimating e.s.d.'s involving l.s. planes.
Refinement. Refinement of *F*^2^ against ALL reflections. The weighted *R*-factor *wR* and goodness of fit *S* are based on *F*^2^, conventional *R*-factors *R* are based on *F*, with *F* set to zero for negative *F*^2^. The threshold expression of *F*^2^ > σ(*F*^2^) is used only for calculating *R*-factors(gt) *etc*. and is not relevant to the choice of reflections for refinement. *R*-factors based on *F*^2^ are statistically about twice as large as those based on *F*, and *R*- factors based on ALL data will be even larger.

### Fractional atomic coordinates and isotropic or equivalent isotropic displacement parameters (Å^2^)

**Table:**

	*x*	*y*	*z*	*U*_iso_*/*U*_eq_	Occ. (<1)
O1	1.02612 (11)	0.7739 (2)	0.65681 (10)	0.1009 (5)	
O2	0.8577 (2)	−0.1440 (4)	1.19028 (19)	0.0963 (8)	0.50
O2'	0.9276 (2)	−0.1336 (4)	1.1217 (2)	0.0830 (8)	0.50
N1	0.73234 (8)	0.68042 (17)	0.96028 (7)	0.0500 (3)	
C1	0.55071 (11)	0.7452 (3)	0.91838 (13)	0.0713 (4)	
H1A	0.5513	0.7464	0.8508	0.107*	
H1B	0.4989	0.8327	0.9318	0.107*	
H1C	0.5381	0.6119	0.9381	0.107*	
C2	0.65035 (10)	0.8149 (2)	0.97202 (11)	0.0573 (4)	
H2A	0.6642	0.9470	0.9495	0.069*	
H2B	0.6473	0.8252	1.0396	0.069*	
C3	0.79421 (9)	0.69945 (19)	0.89373 (9)	0.0459 (3)	
C4	0.79749 (10)	0.8498 (2)	0.82587 (10)	0.0557 (3)	
H4	0.7532	0.9571	0.8198	0.067*	
C5	0.86839 (11)	0.8333 (2)	0.76856 (10)	0.0585 (4)	
H5	0.8715	0.9308	0.7225	0.070*	
C6	0.93648 (10)	0.6734 (2)	0.77748 (9)	0.0523 (3)	
C7	0.93244 (9)	0.5240 (2)	0.84424 (8)	0.0485 (3)	
H7	0.9774	0.4178	0.8501	0.058*	
C8	0.86064 (9)	0.53478 (19)	0.90209 (8)	0.0440 (3)	
C9	0.83555 (9)	0.40894 (19)	0.97708 (8)	0.0452 (3)	
C10	0.75611 (9)	0.5055 (2)	1.01106 (8)	0.0473 (3)	
C11	0.71439 (11)	0.4259 (2)	1.08601 (10)	0.0582 (4)	
H11	0.6627	0.4907	1.1088	0.070*	
C12	0.75280 (12)	0.2485 (2)	1.12461 (10)	0.0626 (4)	
H12	0.7263	0.1923	1.1745	0.075*	
C13	0.83086 (11)	0.1487 (2)	1.09134 (9)	0.0573 (4)	
C14	0.87244 (10)	0.2299 (2)	1.01732 (9)	0.0510 (3)	
H14	0.9244	0.1646	0.9952	0.061*	
C15	1.01501 (12)	0.6612 (3)	0.71893 (11)	0.0666 (4)	
H15	1.0603	0.5559	0.7315	0.080*	
C16	0.87062 (18)	−0.0411 (3)	1.13397 (14)	0.0825 (6)	
H16A	0.9220	−0.0876	1.1043	0.099*	0.50
H16B	0.8377	−0.0871	1.1818	0.099*	0.50

### Atomic displacement parameters (Å^2^)

**Table:**

	*U*^11^	*U*^22^	*U*^33^	*U*^12^	*U*^13^	*U*^23^
O1	0.1167 (11)	0.1034 (10)	0.1002 (9)	−0.0055 (8)	0.0663 (9)	0.0204 (8)
O2	0.128 (2)	0.0828 (17)	0.0829 (16)	−0.0009 (16)	0.0324 (16)	0.0299 (14)
O2'	0.0887 (18)	0.0589 (14)	0.1029 (18)	0.0237 (13)	0.0213 (14)	0.0161 (13)
N1	0.0436 (6)	0.0549 (7)	0.0544 (6)	0.0046 (5)	0.0166 (5)	0.0001 (5)
C1	0.0504 (8)	0.0671 (10)	0.0958 (11)	0.0074 (7)	0.0122 (8)	−0.0025 (9)
C2	0.0514 (8)	0.0556 (8)	0.0688 (8)	0.0064 (6)	0.0212 (6)	−0.0073 (7)
C3	0.0385 (6)	0.0522 (7)	0.0475 (6)	−0.0009 (5)	0.0090 (5)	−0.0009 (5)
C4	0.0505 (7)	0.0547 (8)	0.0619 (8)	0.0052 (6)	0.0103 (6)	0.0085 (6)
C5	0.0575 (8)	0.0622 (9)	0.0573 (7)	−0.0046 (7)	0.0142 (6)	0.0120 (7)
C6	0.0461 (7)	0.0622 (8)	0.0505 (7)	−0.0081 (6)	0.0140 (5)	−0.0001 (6)
C7	0.0404 (6)	0.0550 (8)	0.0509 (6)	−0.0004 (5)	0.0103 (5)	−0.0014 (6)
C8	0.0386 (6)	0.0494 (7)	0.0440 (6)	−0.0013 (5)	0.0078 (5)	−0.0001 (5)
C9	0.0405 (6)	0.0512 (7)	0.0441 (6)	−0.0021 (5)	0.0078 (5)	−0.0012 (5)
C10	0.0429 (6)	0.0535 (7)	0.0461 (6)	−0.0024 (5)	0.0102 (5)	−0.0035 (5)
C11	0.0570 (8)	0.0691 (9)	0.0531 (7)	−0.0048 (7)	0.0218 (6)	−0.0034 (7)
C12	0.0695 (9)	0.0721 (10)	0.0492 (7)	−0.0153 (8)	0.0189 (7)	0.0023 (7)
C13	0.0637 (9)	0.0550 (8)	0.0507 (7)	−0.0098 (7)	0.0040 (6)	0.0046 (6)
C14	0.0483 (7)	0.0521 (8)	0.0519 (7)	−0.0001 (6)	0.0074 (5)	0.0003 (6)
C15	0.0654 (9)	0.0742 (10)	0.0657 (9)	−0.0152 (8)	0.0268 (7)	−0.0035 (8)
C16	0.1016 (15)	0.0660 (11)	0.0722 (11)	−0.0161 (11)	−0.0043 (10)	0.0172 (10)

### Geometric parameters (Å, °)

**Table:**

O1—C15	1.1902 (19)	C6—C7	1.3852 (18)
O2—C16	1.093 (3)	C6—C15	1.466 (2)
O2—H16B	0.4697	C7—C8	1.3842 (17)
O2'—C16	1.029 (3)	C7—H7	0.9300
N1—C3	1.3778 (16)	C8—C9	1.4453 (17)
N1—C10	1.3818 (17)	C9—C14	1.3816 (18)
N1—C2	1.4628 (16)	C9—C10	1.4134 (17)
C1—C2	1.498 (2)	C10—C11	1.3974 (18)
C1—H1A	0.9600	C11—C12	1.370 (2)
C1—H1B	0.9600	C11—H11	0.9300
C1—H1C	0.9600	C12—C13	1.403 (2)
C2—H2A	0.9700	C12—H12	0.9300
C2—H2B	0.9700	C13—C14	1.3896 (19)
C3—C4	1.3990 (18)	C13—C16	1.467 (2)
C3—C8	1.4144 (17)	C14—H14	0.9300
C4—C5	1.371 (2)	C15—H15	0.9300
C4—H4	0.9300	C16—H16A	0.9300
C5—C6	1.404 (2)	C16—H16B	0.9300
C5—H5	0.9300		
			
C3—N1—C10	108.81 (10)	C7—C8—C9	133.70 (12)
C3—N1—C2	126.20 (11)	C3—C8—C9	106.54 (11)
C10—N1—C2	124.89 (11)	C14—C9—C10	119.79 (11)
C2—C1—H1A	109.5	C14—C9—C8	133.94 (12)
C2—C1—H1B	109.5	C10—C9—C8	106.27 (11)
H1A—C1—H1B	109.5	N1—C10—C11	129.17 (12)
C2—C1—H1C	109.5	N1—C10—C9	109.24 (11)
H1A—C1—H1C	109.5	C11—C10—C9	121.58 (13)
H1B—C1—H1C	109.5	C12—C11—C10	117.24 (13)
N1—C2—C1	112.52 (12)	C12—C11—H11	121.4
N1—C2—H2A	109.1	C10—C11—H11	121.4
C1—C2—H2A	109.1	C11—C12—C13	122.20 (13)
N1—C2—H2B	109.1	C11—C12—H12	118.9
C1—C2—H2B	109.1	C13—C12—H12	118.9
H2A—C2—H2B	107.8	C14—C13—C12	120.13 (13)
N1—C3—C4	129.60 (12)	C14—C13—C16	118.77 (16)
N1—C3—C8	109.13 (11)	C12—C13—C16	121.09 (15)
C4—C3—C8	121.26 (12)	C9—C14—C13	119.05 (13)
C5—C4—C3	117.61 (13)	C9—C14—H14	120.5
C5—C4—H4	121.2	C13—C14—H14	120.4
C3—C4—H4	121.2	O1—C15—C6	125.94 (17)
C4—C5—C6	121.85 (13)	O1—C15—H15	117.0
C4—C5—H5	119.1	C6—C15—H15	117.0
C6—C5—H5	119.1	O2'—C16—C13	133.0 (3)
C7—C6—C5	120.34 (12)	O2—C16—C13	138.7 (3)
C7—C6—C15	118.06 (13)	O2—C16—H16A	110.6
C5—C6—C15	121.58 (13)	C13—C16—H16A	110.6
C8—C7—C6	119.15 (12)	O2'—C16—H16B	113.5
C8—C7—H7	120.4	C13—C16—H16B	113.5
C6—C7—H7	120.4	H16A—C16—H16B	135.8
C7—C8—C3	119.75 (11)		
			
C3—N1—C2—C1	94.54 (16)	C3—N1—C10—C11	179.12 (13)
C10—N1—C2—C1	−81.23 (17)	C2—N1—C10—C11	−4.5 (2)
C10—N1—C3—C4	179.26 (13)	C3—N1—C10—C9	0.26 (14)
C2—N1—C3—C4	2.9 (2)	C2—N1—C10—C9	176.66 (11)
C10—N1—C3—C8	−0.76 (13)	C14—C9—C10—N1	−179.99 (11)
C2—N1—C3—C8	−177.10 (12)	C8—C9—C10—N1	0.34 (13)
N1—C3—C4—C5	179.09 (13)	C14—C9—C10—C11	1.04 (18)
C8—C3—C4—C5	−0.88 (19)	C8—C9—C10—C11	−178.63 (11)
C3—C4—C5—C6	−0.7 (2)	N1—C10—C11—C12	−179.57 (12)
C4—C5—C6—C7	1.2 (2)	C9—C10—C11—C12	−0.83 (19)
C4—C5—C6—C15	−177.23 (13)	C10—C11—C12—C13	0.1 (2)
C5—C6—C7—C8	−0.16 (19)	C11—C12—C13—C14	0.5 (2)
C15—C6—C7—C8	178.34 (12)	C11—C12—C13—C16	179.97 (14)
C6—C7—C8—C3	−1.36 (17)	C10—C9—C14—C13	−0.47 (18)
C6—C7—C8—C9	179.95 (13)	C8—C9—C14—C13	179.09 (13)
N1—C3—C8—C7	−178.05 (11)	C12—C13—C14—C9	−0.2 (2)
C4—C3—C8—C7	1.92 (18)	C16—C13—C14—C9	−179.78 (12)
N1—C3—C8—C9	0.96 (13)	C7—C6—C15—O1	177.41 (16)
C4—C3—C8—C9	−179.07 (12)	C5—C6—C15—O1	−4.1 (2)
C7—C8—C9—C14	−1.6 (2)	C14—C13—C16—O2'	1.6 (4)
C3—C8—C9—C14	179.62 (13)	C12—C13—C16—O2'	−177.9 (3)
C7—C8—C9—C10	178.03 (13)	C14—C13—C16—O2	179.8 (3)
C3—C8—C9—C10	−0.78 (13)	C12—C13—C16—O2	0.3 (4)
